# Metabolic Activation of Flavin Monooxygenase-mediated Trimethylamine-*N*-Oxide Formation in Experimental Kidney Disease

**DOI:** 10.1038/s41598-019-52032-9

**Published:** 2019-11-04

**Authors:** Alexander J. Prokopienko, Raymond E. West, Daniel P. Schrum, Jason R. Stubbs, François A. Leblond, Vincent Pichette, Thomas D. Nolin

**Affiliations:** 1 Center for Clinical Pharmaceutical Sciences, Department of Pharmaceutical Sciences or Department of Pharmacy and Therapeutics, School of Pharmacy, University of Pittsburgh, Pittsburgh, PA United States; 2 The Kidney Institute, and Department of Internal Medicine, Division of Nephrology & Hypertension, University of Kansas Medical Center, Kansas City, KS United States; 3 ProMetic Life Sciences Inc., Laval, Québec Canada; 4 Service de Néphrologie et Centre de Recherche, Hôpital Maisonneuve-Rosemont, Département de Pharmacologie, Université de Montréal, Montréal, Québec Canada

**Keywords:** Cardiovascular diseases, End-stage renal disease

## Abstract

Cardiovascular disease (CVD) remains the leading cause of death in chronic kidney disease (CKD) patients despite treatment of traditional risk factors, suggesting that non-traditional CVD risk factors are involved. Trimethylamine-*N*-oxide (TMAO) correlates with atherosclerosis burden in CKD patients and may be a non-traditional CVD risk factor. Serum TMAO concentrations are significantly increased in CKD patients, which may be due in part to increased hepatic flavin monooxygenase (FMO)-mediated TMAO formation. The objective of this work was to elucidate the mechanism of increased FMO activity in CKD. In this study, FMO enzyme activity experiments were conducted *in vitro* with liver microsomes isolated from experimental CKD and control rats. Trimethylamine was used as a probe substrate to assess FMO activity. The FMO activator octylamine and human uremic serum were evaluated. FMO gene and protein expression were also determined. FMO-mediated TMAO formation was increased in CKD versus control. Although gene and protein expression of FMO were not changed, metabolic activation elicited by octylamine and human uremic serum increased FMO-mediated TMAO formation. The findings suggest that metabolic activation of FMO-mediated TMAO formation is a novel mechanism that contributes to increased TMAO formation in CKD and represents a therapeutic target to reduce TMAO exposure and CVD.

## Introduction

Cardiovascular disease (CVD) events are the leading cause of death in chronic kidney disease (CKD) patients despite aggressive treatment of traditional risk factors^1^, suggesting that non-traditional CVD risk factors may play an important role^2,3^. Trimethylamine-*N*-oxide (TMAO) promotes atherosclerosis in preclinical models^4^, is elevated and associated with CVD in CKD patients^5–7^, and may be a novel non-traditional CVD risk factor^8^. For instance, we have shown that circulating TMAO concentrations are 30-fold higher in patients with end-stage kidney disease (ESKD) than in the general population and correlate with coronary atherosclerosis burden^9^. Elevated TMAO concentrations may also directly contribute to fibrosis and non-ischemic heart failure in CKD^10–12^. TMAO concentrations increase disproportionately from Stage 4 to 5 CKD (i.e., 20 μM to 94 μM) compared to Stage 1 to 3 CKD (i.e., 3.3 μM to 10 μM)^7,9^, strongly implicating formation of TMAO as a contributor to elevated serum TMAO concentrations in advanced CKD. Recently, we demonstrated that enhanced hepatic flavin monooxygenase (FMO)-mediated TMAO formation along with decreased renal clearance contributes to increased TMAO exposure in a mouse model of experimental kidney disease^13^. However, the mechanism driving increased TMAO formation remained unclear.

Mechanistic evaluation of increased TMAO formation could shed light on novel therapeutic strategies for the prevention of CVD in CKD. In fact, recent studies provide proof of principle that FMO activity is a potential therapeutic target^14^. In mice, antisense oligonucleotide-mediated knockdown of *FMO3* (the primary isoform responsible for TMAO formation in humans) leads to decreases in serum TMAO concentrations and atherosclerosis formation^15,16^. Therapeutically targeting FMOs may be particularly effective in the setting of increased FMO-mediated TMAO formation as seen in diabetes and CKD^13,15,17^.

The objective of this study was to elucidate potential mechanisms of increased hepatic FMO-mediated TMAO formation observed in CKD. We accomplished this by conducting FMO enzyme activity experiments with CKD and control rat microsomal fractions. We also investigated potential changes in mRNA and protein expression of FMOs.

## Results

### Characteristics of CKD and control rats

**Figure 1:**
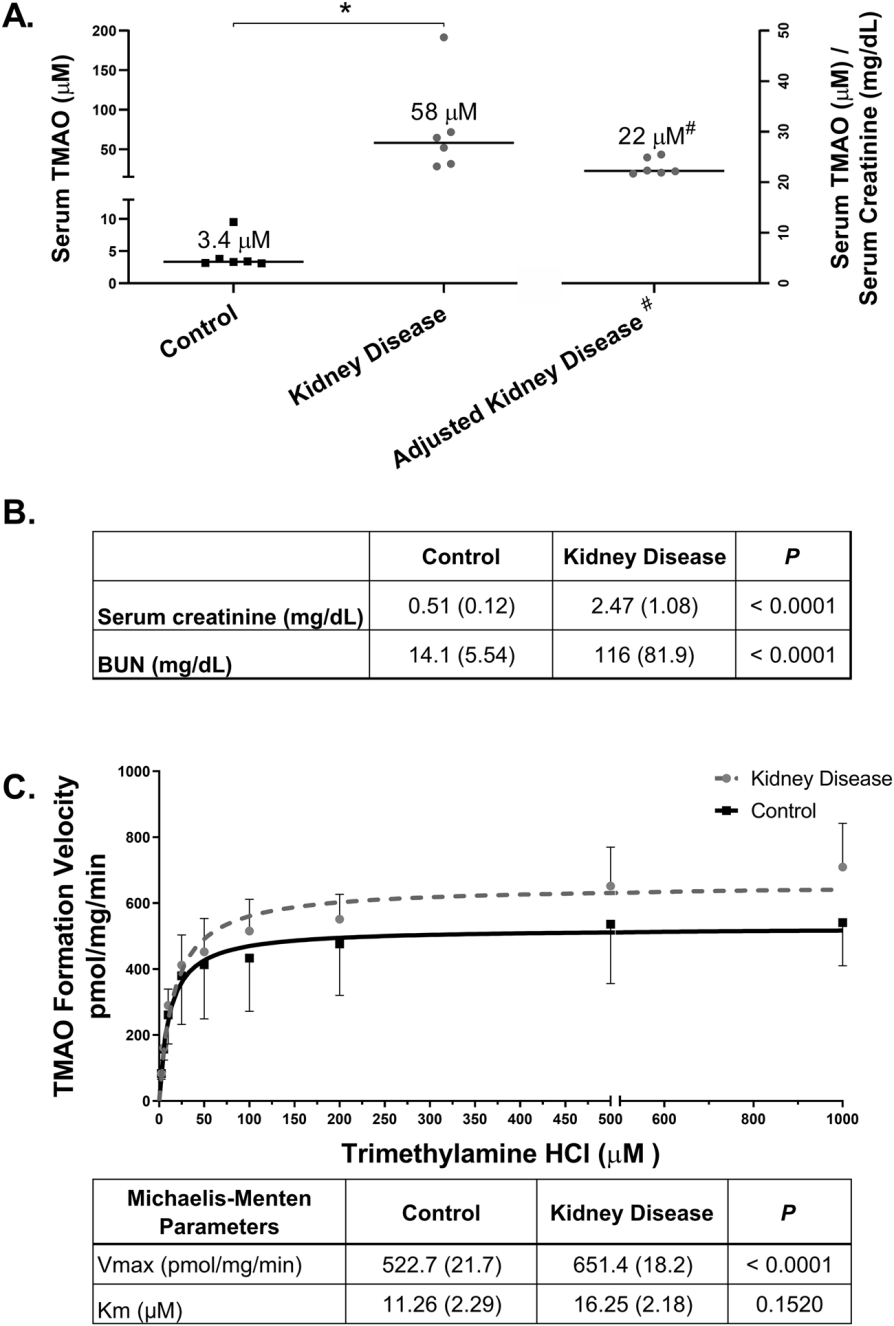
FMO Activity and Serum TMAO Concentrations in CKD and Control Rats. (**A**) Serum concentrations of TMAO compared between CKD (n = 6) rats and controls (n = 6). ^#^Serum TMAO concentrations were adjusted for kidney function by dividing individual TMAO concentrations by the corresponding serum creatinine (mg/dL) values. *P < 0.05 for CKD compared to control with a Mann-Whitney test. (**B**) Biochemical characteristics were compared between CKD (n = 12) and controls (n = 12) with a Student’s *t*-tests and presented as mean ± SD. (**C**) Michaelis-Menten plot for the formation of TMAO in rat liver microsomes of both control and CKD rats. Liver microsomal protein (0.5 mg/mL) was incubated with various concentrations of trimethylamine (2.5–1000 µM) for 60 min at 37 °C. Each point represents the mean ± SEM of 6 rats in each group. The parameters were statistically compared with an F-test.

TMAO exposure was compared between CKD and control rats. The median (interquartile range) TMAO concentration in CKD versus control serum was 58 µM (31–102) and 3.4 µM (3.15–5.24), respectively (*P* = 0.0022; Fig. 1A). Serum creatinine and blood urea nitrogen (BUN) were also higher in CKD rats versus control (*P* < 0.0001; Fig. 1B).

### FMO activity in CKD and control rats

The potential mechanism of altered FMO-mediated TMAO formation in CKD was assessed by comparing enzyme kinetic parameters observed in CKD rat liver microsomes with control rat microsomes. The V_max_ (i.e., the maximal rate at which the enzyme catalyzes the reaction) for TMAO formation was increased by 25% in CKD versus control (651.4 ± 18 versus 522.7 ± 22 pmol/mg protein/minute, *P* < 0.0001; Fig. 1C). The K_m_ value (i.e., substrate concentration at half the maximum velocity) was not different between groups (16.3 ± 2.3 versus 11.3 ± 2.5 μM, *P* = 0.15).

### Gene and protein expression

**Figure 2:**
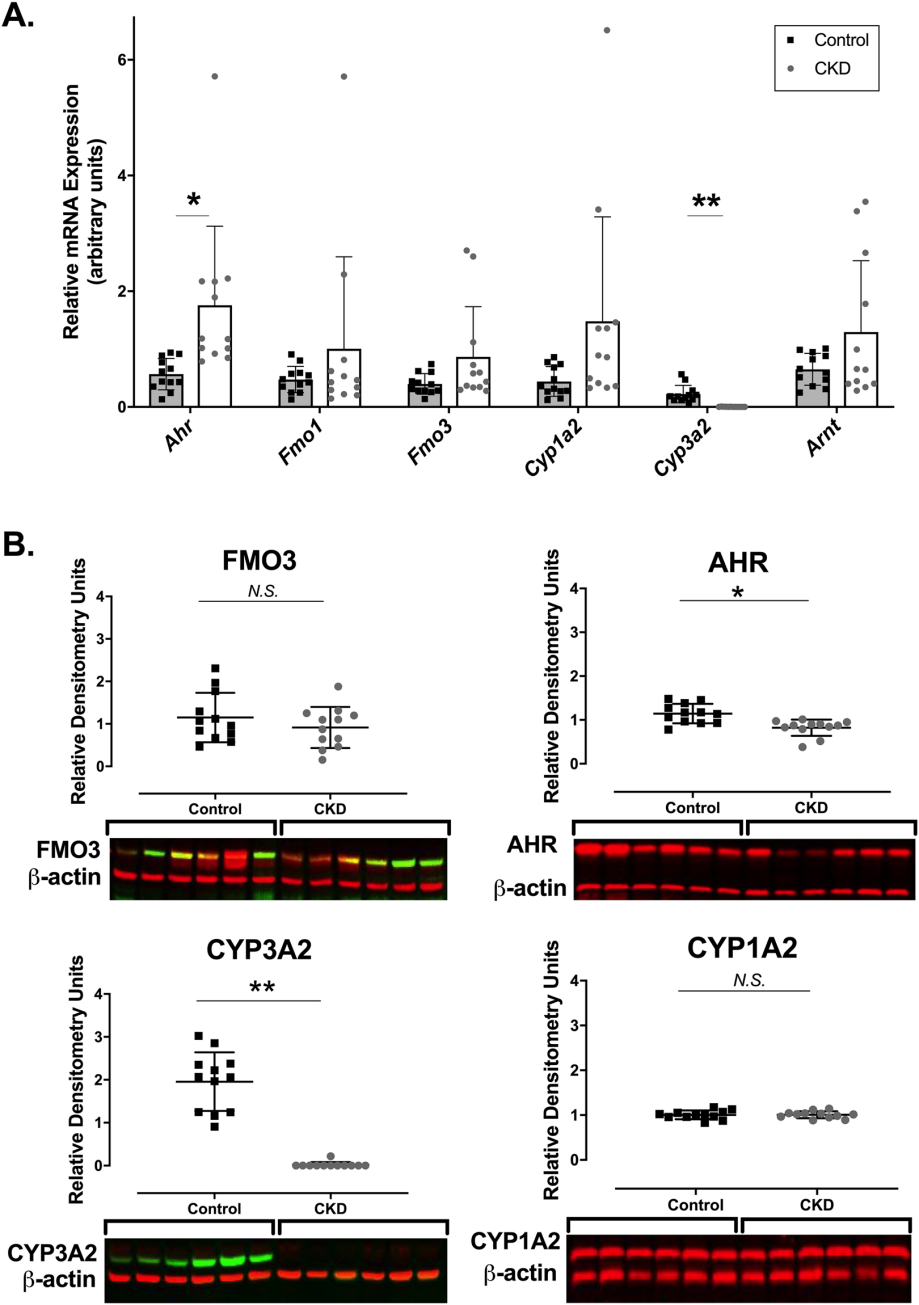
mRNA and Protein Expression. (**A**) mRNA expression of hepatic drug metabolism enzymes and related genes (*Fmo3*, *Fmo1*, *Arnt*, *Ahr*, *Cyp1a2*, *Cyp3a2* and *Actb*) in control and CKD rats. Hepatic mRNA levels were normalized to β-Actin and expressed relative to the controls using absolute quantification. Experiments were conducted in duplicates, and results are presented as mean ± SD of 12 rats in each group. *P < 0.05 and **P < 0.001 compared with control by Student’s *t*-tests. (**B**) Protein expression of drug metabolism enzymes and related proteins (FMO3, AhR, CYP1A2, CYP3A2 and β-Actin) in control and CKD rat livers. The densitometry units of protein expression were normalized to that of β-Actin. The results are presented as mean ± SD of 12 rats in each group. The lower panel represents blots of six control and six CKD rats. *P < 0.05 and **P < 0.001 compared with control by Student’s *t*-tests. The corresponding full-length blo*t*s are presented in Supplementary Figures 2–5.

In order to evaluate whether any changes in FMO activity were due to changes in gene or protein regulation, hepatic FMO mRNA and protein expression were assessed (Fig. 2A). No change in *Fmo1* or *Fmo3* mRNA was observed in CKD versus control. The positive control *Cyp(Cytochrome P450)-3a2* was downregulated in CKD versus control (*P* < 0.0001). *Ahr* (aryl-hydrocarbon receptor) was upregulated in CKD versus control (*P* < 0.05) but *Arnt* (aryl hydrocarbon receptor nuclear translocator) and *Cyp1a2* were not.

FMO3, CYP1A2, AhR and CYP3A2 protein expression is presented in Fig. 2B. Decreases in AhR (*P* < 0.01) and CYP3A2 (*P* < 0.001) but no change in FMO3 and CYP1A2 protein expression were observed in CKD versus control.

### Metabolic activation and inhibition of FMO

**Figure 3:**
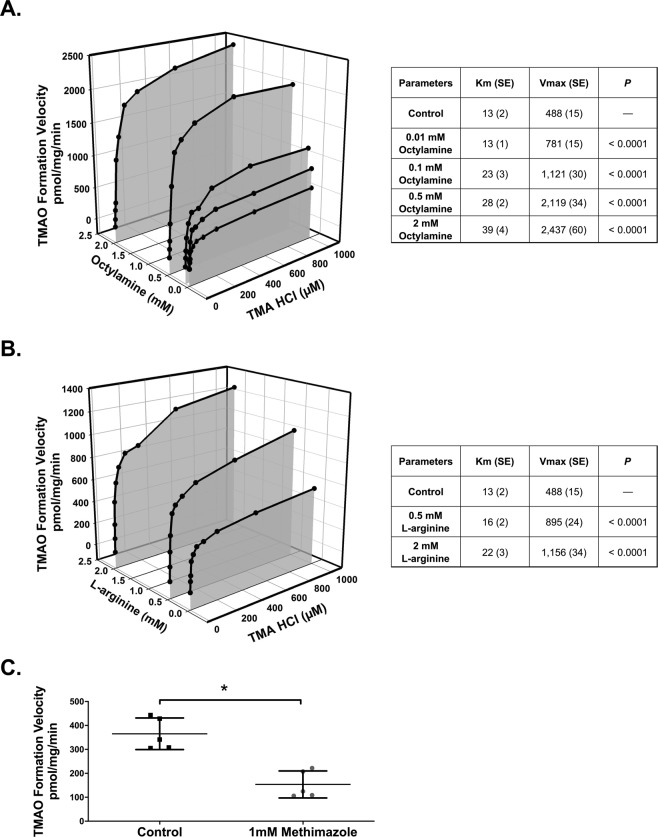
Metabolic Activation and Inhibition of FMO. Michaelis-Menten plot for the formation of TMAO in rat liver microsomes in the presence of increasing concentrations of (**A**) octylamine and (**B**) L-arginine. Liver microsomal protein (0.5 mg/mL) was incubated with various concentrations of trimethylamine (2.5–1000 µM) for 60 min at 37 °C in the presence of either octylamine (0.01–2 mM) or L-arginine (0.5 and 2 mM). Each point represents the mean of at least 3 replicates at each trimethylamine concentration in each group. The parameters were statistically compared with an F-test. Note that *P* value represents a comparison of Vmax for each octylamine or L-arginine concentration versus control. (**C**) FMO-mediated TMAO formation was also assessed in the presence of the FMO inhibitor methimazole. Liver microsomal protein (0.5 mg/mL) was incubated with 50 µM of trimethylamine for 60 min at 37 °C in the presence of 1 mM of methimazole. Each point represents the mean ± SD of 5 replicates. *P < 0.001for treatment group compared to control by Student’s *t*-test.

**Figure 4:**
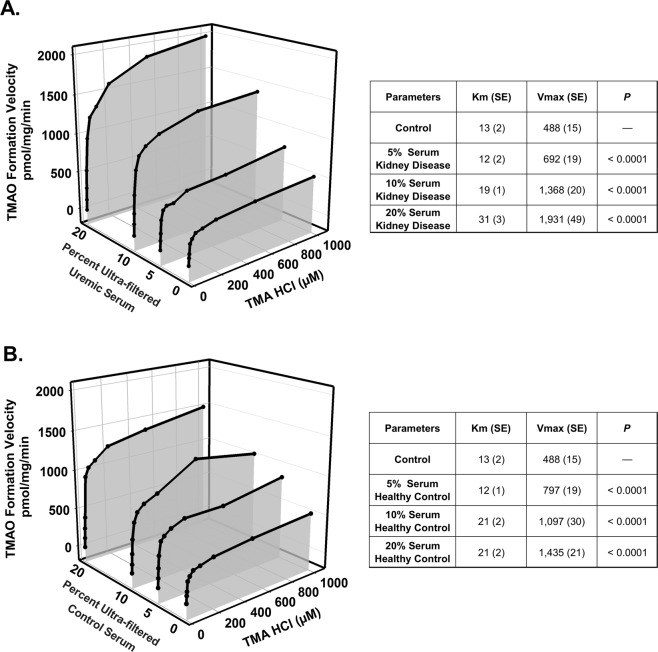
Metabolic Activation of FMO-mediated TMAO Formation by Human Serum. Michaelis-Menten plots for the formation of TMAO in rat liver microsomes in the presence of increasing percentages of (**A**) human uremic serum and (**B**) healthy control serum. Liver microsomal protein (0.5 mg/mL) was incubated with various concentrations of trimethylamine (2.5–1000 µM) for 60 min at 37 °C in the presence of serum (5–20%). Each point represents the mean of at least 3 replicates at each trimethylamine concentration in each group. The parameters were statistically compared with an F-test. Note that *P* value represents a comparison of Vmax for each percent ultra-filtered serum group versus control.

An atypical kinetic reaction known as metabolic activation was explored as a potential mechanism of increased FMO activity. Concentration-dependent increases of up to 4-fold in metabolic activation were observed with octylamine as a positive activation control (*P* < 0.0001; Fig. 3A). L-arginine was evaluated to identify potential representative endogenous compounds responsible for activation; up to a 1.4-fold increase in metabolic activation was observed in the presence of L-arginine (P < 0.0001; Fig. 3B). FMO-mediated TMAO formation was decreased by 58% (*P* < 0.0001; Fig. 3C) in the presence of the FMO inhibitor methimazole. Lastly, FMO activity was determined in the presence 5–20% ultra-filtered human serum to assess whether solutes retained in ESKD can activate FMO enzymes. TMAO formation velocity was increased up to 3-fold with uremic serum compared to 1.9-fold with healthy control serum (*P* < 0.0001; Fig. 4).

## Discussion

We show for the first time that metabolic activation of hepatic FMOs leads to increased formation of the non-traditional CVD risk factor TMAO, which may contribute to dramatically elevated serum concentrations in CKD rats. These findings corroborate our clinical observations of significantly elevated systemic TMAO concentrations in patients with advanced CKD and provide a novel mechanism for our recent observations of enhanced FMO-mediated TMAO formation in experimental CKD^9,13^.

Mechanistically, metabolic activation of FMO enzymes by uremic solutes may contribute to increased TMAO formation in CKD. In fact, metabolic activation likely contributes to the increased systemic exposure of TMAO observed in CKD, evidenced by disproportionate increases of serum TMAO in advanced CKD relative to earlier stages of CKD. For instance, TMAO serum concentrations are increased 16-fold in CKD rats (Fig. 1B), and 30-fold in ESKD patients compared to controls^9^. The V_max_ of TMAO formation was increased by 25% (*P* < 0.0001; Fig. 1A) in CKD versus control tissue in the isolated microsomal incubation experiments. However, no changes in FMO gene or protein expression (Fig. 2A,B) were observed, suggesting that increased FMO activity was unrelated to changes in expression. The latter finding is inconsistent with our previous observations in mice, in which gene expression changes may partially explain differences in FMO activity^13^, but is not unexpected given well-known species differences in FMO3 regulation in mice compared to rats and humans^18^. For instance, FMO3 expression is also profoundly decreased by testosterone in mice but this effect is less clear in rats and humans^19,20^. Therefore, the current experimental rat model may better reflect FMO3 regulation and metabolic activity observed in humans with CKD. Furthermore, changes in FMO3 mRNA and protein expression often do not correlate with serum TMAO exposure, suggesting that alternative mechanisms such as metabolic activation may regulate FMO activity^21^. Nevertheless, current and previous findings collectively indicate that FMO-mediated TMAO formation is increased in CKD, and our present data suggest that it is mechanistically driven by metabolic activation.

Metabolic activation was elicited with octylamine, L-arginine, and uremic and healthy control serum. The up to 4-fold increase in V_max_ in the presence of octylamine clearly demonstrates that FMO-mediated TMAO formation can be activated (Fig. 3A)^14^. The increased metabolic activation with human uremic serum versus healthy control serum suggests that increased concentrations of uremic solutes retained in uremic serum elicit greater metabolic activation (Fig. 4A). Although the potential effector compounds remain unknown, the endogenous solute L-arginine elicits activation (Fig. 3B), and other structurally similar endogenous substances may behave in a similar manner. Interestingly, L-arginine serum concentrations are similar in CKD and healthy patients^22^, and it is possible that L-arginine is contributing to the metabolic activation observed with healthy control serum (Fig. 4B).

Metabolic activation is an atypical kinetic reaction. Activation of FMO-mediated TMAO formation indicates that ‘effector’ compounds are eliciting structural or electrostatic changes in the FMO catalytic site^23^. In fact, FMO enzymes can be activated by a broad range of effector compounds that are known uremic retention solutes, including primary amines, guanidine derivatives and small peptides^14,24,25^. In CKD, these compounds may deposit and accumulate in liver tissue, specifically in the microsomal fraction where they can act as FMO effectors^26,27^, and this may explain the present findings of metabolic activation and increased TMAO formation in experimental CKD^28–32^.

In accordance with enzyme kinetic principles, the increased V_max_ resulting from metabolic activation of FMOs would result in a corresponding increase in TMAO formation when substrate (trimethylamine) concentrations exceed the relatively low K_m_ value (enzyme affinity for substrate) of approximately 28 µM for FMO3 enzymes^33^. In this scenario, the reaction is rate limited and not substrate/supply limited, such that any increase in V_max_ leads to a corresponding increase in TMAO formation. Trimethylamine concentrations can exceed this K_m_ value in humans considering trimethylamine production is approximately 50 mg/day, especially after a precursor-nutrient rich meal^34–36^. Furthermore, FMO3 gene polymorphisms are associated with increased TMAO concentrations, indicating that FMO-mediated TMAO formation is not supply limited^17^. Overall, these data support a novel biologically plausible mechanism involving metabolic activation that increases the V_max_ of FMO-mediated TMAO formation and contributes to well documented increases in systemic concentrations of TMAO in CKD patients.

Although activation of FMOs leads to increased TMAO formation in this experimental model of CKD, this requires validation in humans. We and several other investigators have reported elevated concentrations of trimethylamine and/or TMAO in CKD patients (Supplementary Table S1)^5,7,9,37^, but to date the potential mechanisms are unclear. Decreased renal clearance undoubtedly contributes to elevated serum TMAO concentrations^38^, although the relative contribution is unclear and likely minimal in the setting of advanced kidney disease. Additionally, the observations that systemic TMAO concentrations increase disproportionately in advanced CKD relative to earlier stages of CKD^9^, and that hemodialysis is relatively ineffective at lowering systemic exposure of TMAO (i.e., evidenced by pre-dialysis concentrations)^39^, despite high intradialytic clearance^37,40^, support the premise that TMAO formation is increased in CKD. It has been postulated that TMAO production is not changed in ESKD patients receiving chronic hemodialysis, and that the extraordinarily increased concentrations in these patients are due partly to the inability of hemodialysis to provide clearances of the magnitude achieved by tubular secretion^40^. While the latter point is valid, TMAO concentrations rise disproportionately even in non-dialyzed Stage 4 CKD patients with residual kidney function and presumably residual secretory clearance. One possible explanation is that the loss of secretory clearance in ESKD is outweighed by a simultaneous increase in TMAO production leading to the increased systemic exposure observed. Lastly, though decreased nonrenal clearance also may impact the systemic exposure of substrates predominantly cleared by the pathway in question^41^, TMAO undergoes little nonrenal clearance^42^, so this is unlikely to explain the increases in systemic exposure observed as kidney disease progresses. Overall, high systemic TMAO exposure is likely due to a combination of decreased renal clearance^43^, and increased TMAO production in kidney disease.

There are several limitations of the current study. Post-translational modifications like phosphorylation may affect enzyme activity and this was not evaluated. The microbiome plays an integral role in the production of trimethylamine, the FMO substrate that is metabolized into TMAO, and we did not explore how it is changed in CKD or whether any changes are associated with altered TMAO formation. It is also possible that the potential effector compounds may be derived from dietary nutrients. The patients that contributed the uremic and healthy serum were not on a controlled diet, which may have influenced the metabolic activation results. In addition, serum from other donors was not evaluated to replicate these findings. There are hundreds of known uremic retention solutes that accumulate in CKD and future work is necessary to determine the potential effector compounds that may activate FMO-mediated TMAO formation^44^. Future *in vitro* and *in vivo* studies will evaluate FMO enzyme activity in the presence of individual solutes (i.e., TMAO, urea, primary amines, guanidine derivatives, etc.). Lastly, therapeutically targeting FMO3 function by partial inhibition may not induce the undesirable symptoms of trimethylaminuria observed in patients with inactive FMO3 enzymes^35^, but this should be carefully evaluated.

In conclusion, we show for the first time that metabolic activation of hepatic FMOs leads to increased formation of the non-traditional CVD risk factor TMAO. These data provide important mechanistic insight into the function of hepatic FMOs, as metabolic activation may contribute to the elevated TMAO concentrations observed as kidney function declines. FMO-mediated metabolism may be a therapeutic target to decrease TMAO exposure and thereby lower rates of CVD in patients with CKD.

## Methods

### Chemical reagents

Trimethylamine hydrochloride, TMAO, NADPH, magnesium chloride, tris (hydroxymethyl) aminomethane (Trizma® base), Trizma® hydrochloride, n-octylamine, methimazole, L-arginine and formic acid ( ≥ 95%) were purchased from Sigma-Aldrich (St. Louis, MO). Deuterated internal standard (*d*9-trimethylamine N-oxide) was purchased from Cambridge Isotopes (Cambridge, MA, USA). Optima LC–MS grade water, acetonitrile, and methanol was purchased from Fisher Scientific (Pittsburgh, PA). Taqman® primers used for mRNA quantification were purchased from Applied Biosystems (Foster City, CA). All fluorescent antibodies were purchased from Abcam (Cambridge, MA) (Codes: ab2769, ab8226, ab22717, ab126790, ab195627, ab186693 and ab175774).

### Experimental model

Male Sprague-Dawley rats (Charles River, Saint-Charles, PQ, Canada) that weighed 200 to 300 g were fed standard rat chow and water *ad libitum* on a 12-hour light/dark cycle. Control rats were pair-fed matching amounts of standard rat chow consumed by CKD rats. The Canadian Council on Animal Care guidelines were observed for care and use of laboratory animals. The experimental protocol was authorized by the Maisonneuve-Rosemont Hospital Research Centre Animal Care Committee. Experimental CKD was surgically induced by first performing a 2/3rd nephrectomy of the left kidney followed 7 days later by a complete right nephrectomy, as previously described^45^. Control rats underwent to two sham laparotomies. Rats were sacrificed 42 days after the initial surgery and livers were immediately harvested and stored at −80 °C.

### Determination of FMO activity

Metabolic activity of hepatic FMOs was assessed with isolated microsomes of control (n = 6) and CKD (n = 6) rat livers. Specifically, trimethylamine was used as a probe substrate of FMO enzymes, and formation rate of TMAO was used as a surrogate measurement of FMO activity. Hepatic microsomes (i.e., the liver fraction containing FMOs) were isolated by differential ultra-centrifugation as previously described^46^. Incubation times and microsomal protein concentrations were optimized to achieve linear formation of TMAO in the experiments. Microsomal incubations using 0.5 mg/mL of microsomal protein in 0.02 M Tris-HCl buffer (pH 7.4) containing 1 mM nicotinamide adenine dinucleotide phosphate (NADPH), as an essential cofactor, and 5 mM magnesium chloride (MgCl_2_) were conducted for each rat. The microsomal incubations were pre-warmed in the presence of NADPH for 3 minutes at 37 °C. To start the reaction, 3 μL of increasing concentrations of trimethylamine (2.5, 5, 10, 25, 50, 100, 200, 500, 1000 μM) was added, and microsomes were incubated for 60 min at 37 °C. Final reaction volumes were 300 μL. Each trimethylamine incubation experiment (i.e., each concentration) was conducted in duplicate (CKD vs. healthy control). Negative controls omitting NADPH and trimethylamine were assessed for all incubations. Reactions were stopped by adding 300 μL of ice-cold methanol. TMAO was quantified by ultra-performance liquid chromatography tandem mass spectrometry (LC-MS/MS) as we reported previously^47^.

### mRNA analysis

RNA isolation and real-time quantitative polymerase chain reaction (RT-qPCR) were conducted as recommended by MIQE guidelines^48^. Total RNA was extracted from homogenized liver tissue from n = 12 CKD and n = 12 control rats, using QiaShredder and RNeasy Mini Kit (Qiagen, Valencia, CA). RNA purity (260/280 ratios ranging from 1.8 to 2.0) and concentrations were determined by measuring the optical density at 260 nm and 280 nm using NanoDrop (ThermoFisher Scientific, Waltham, MA). cDNA was then prepared using SuperScript III reverse transcriptase (Invitrogen, San Diego, CA) with 1 μg of total RNA and random hexamers. *Fmo3*, *Fmo1*, *Arnt*, *Ahr*, *Cyp1a2*, *Cyp3a2* and *Actb* (β-Actin) genes were quantified by RT-qPCR with Applied Biosystems 7500 Fast Real-Time PCR System (Foster City, CA) using Taqman® Gene Expression Master Mix (Applied Biosystems, Foster City, CA) and Taqman® specific primers. Template and reverse transcriptase controls were not included in every reaction. *Ahr* and *Arnt* were selected because the aryl-hydrocarbon receptor (AhR) signaling pathway may be activated in CKD and partially regulate FMO expression^49,50^. Hepatic mRNA concentrations were normalized to *Actb* and expressed relative to the controls using absolute quantification.

### Western blot analysis

The protein expression of hepatic FMO3, CYP1A2, CYP3A2, AHR and β-Actin was determined with fluorescent Western blotting. Homogenized liver tissue from n = 12 CKD and n = 12 control rats (30 μg total protein) were separated by 4–15% Mini Protean TGX (Bio-Rad, Hercules, CA) gel electrophoresis and transferred onto PVDF membranes. Transferred membranes were blocked for 1 hour at room temperature using Odyssey Blocking Buffer. Membranes were incubated at 4 °C overnight with specific primary antibodies diluted in 50:50 blocking buffer and TBS- 0.1% Tween (TBST) (1:1000–2500 mouse monoclonal anti- β-Actin, CYP1A2 and AhR; 1:1000 polyclonal rabbit anti- FMO3 and CYP3A2). Membranes were washed four times for 10 minutes with TBST and then for one hour with 50:50 blocking buffer and TBST. Membranes were then incubated for 1 hour in fluorescent secondary antibodies at room temperature (1:20000 donkey anti-rabbit; or 1:10000 donkey anti-mouse). Finally, membranes were washed with TBST, and antibody binding was measured by a LI-COR fluorescent reader (Lincoln, NE) detection system. CYP1A2 and CYP3A2 were selected as controls because their expression in experimental CKD is unchanged and decreased, respectively^51^. Specificity was assessed by incubating blots that were loaded with control homogenized rat tissue with each primary antibody to check for overlapping fluorescent bands (see Supplementary S-Fig. 1). The linear range of detection was determined for a range of total protein on these blots. Band intensity was quantified by densitometry using ImageJ software and normalized to β-Actin expression.

### Assessment of FMO metabolic activation and inhibition

Metabolic activation was explored as a potential mechanism of increased FMO activity. Metabolic activation was assessed using the experimental conditions described above for the determination of FMO activity. However, male Sprague-Dawley rat liver microsomes (purchased from Sekisui XenoTech, Kansas City, KS) were used in these experiments. The reference point of FMO activity in these microsomes was consistent with the experimental control rats. Also, potential activating compounds (0.01–2 mM octylamine or 0.5 and 2 mM L-arginine), and human serum were added to the microsomal incubations prior to the start of the reaction. The FMO inhibitor methimazole (1 mM) was added to 5 replicates of the 50 μM trimethylamine incubates. TMAO formation rates were assessed to measure changes in FMO metabolic activity.

In experiments with human serum, appropriate volumes were added to achieve the 5, 10 and 20% of total reaction volume. Baseline concentrations of trimethylamine and TMAO in the human serum were quantified before starting the incubation reactions (Supplementary Table S1). Final TMAO formation was calculated by taking the final TMAO concentration minus the baseline TMAO concentration. Concentrations of baseline trimethylamine in serum were below the limit of quantification, and therefore were negligible (Supplementary Table S1). Baseline TMAO did not undergo any quantifiable metabolism or retro-reduction into trimethylamine in the microsomal incubations. Healthy human control serum was purchased from a local blood bank and serum from a hemodialysis patient (pre-dialysis sample; serum creatinine, 14.6 mg/dL; BUN, 77 mg/dL) was obtained with informed consent and approval from the University of Kansas Medical Center Institutional Review Board. All serum was ultra-filtered using 10 Kda Satorius Vivaspin® spin columns.

### Data and statistical analysis

Individual rat serum TMAO concentrations were adjusted based on kidney function by dividing by the corresponding serum creatinine values. The formation rate of TMAO was determined from LC-MS/MS quantified TMAO concentrations in the microsomal incubates. Non-linear regression Michaelis-Menten kinetic models were used to fit FMO-mediated TMAO formation data, and the maximum velocity (V_max_) and the affinity constant (K_m_) were estimated and compared by extra-sum-of-squares F-tests with GraphPad Prism (Version 8.0.2; San Diego, CA). Hepatic mRNA levels were normalized to β-Actin and expressed relative to the controls using absolute quantification. Densitometry units of protein expression were normalized to that of β-Actin. Gene and protein expression were not performed in one control rat liver sample due to limited tissue quantity. Student’s *t*-test or Mann-Whitney test for data exhibiting non-normal distribution were used to compare CKD to control rats. *P*-values of < 0.05 were considered significant. All results are presented as mean ± SD, unless otherwise stated.

## Supplementary information

Supplemental Information

## Data Availability

The datasets generated during and/or analyzed during the current study are available from the corresponding author on reasonable request.
