# Supplementary material for: Metabolic Activation of Flavin Monooxygenase-mediated Trimethylamine-N-Oxide Formation in Experimental Kidney Disease
Source: Sci Rep. 2019 Nov 4;9:15901. doi: 10.1038/s41598-019-52032-9 (PMC6828678; doi:10.1038/s41598-019-52032-9)
Supplement: Supplementary file 1 — Supplemental Information [file 41598_2019_52032_MOESM1_ESM.docx]

**Metabolic Activation of Flavin Monooxygenase-mediated Trimethylamine-*N*-Oxide Formation in Experimental Kidney Disease**

**Alexander J. Prokopienko^1^, Raymond E. West III^1^, Daniel P. Schrum^1^, Jason R. Stubbs^2^, François A. Leblond^3^, Vincent Pichette^4^, and Thomas D. Nolin^1^***

^1^ Center for Clinical Pharmaceutical Sciences, Department of Pharmaceutical Sciences or Department of Pharmacy and Therapeutics, School of Pharmacy, University of Pittsburgh, Pittsburgh, PA, United States.

^2^ The Kidney Institute, and Department of Internal Medicine, Division of Nephrology & Hypertension, University of Kansas Medical Center, Kansas City, KS.

^3^ ProMetic Life Sciences Inc., Laval, Québec, Canada.

^4^ Service de Néphrologie et Centre de Recherche, Hôpital Maisonneuve-Rosemont, Département de Pharmacologie, Université de Montréal, Montréal, Québec, Canada.

***Corresponding Author**: Thomas D. Nolin, PharmD, PhD

Department of Pharmacy and Therapeutics

University of Pittsburgh School of Pharmacy

208 Salk Pavilion

335 Sutherland Drive

Pittsburgh, PA, 15261

Tel: (412) 624-4683

E-mail: [nolin@pitt.edu](mailto:nolin@pitt.edu)

**SUPPLEMENTARY INFORMATION**

**Table S1. Trimethylamine (TMA) and TMAO Concentrations in Human Serum Used in Microsomal Incubations**

| Sample | TMA (µg/mL)  No Incubation | TMA (µg/mL)  After Incubation | TMAO (µg/mL)  No Incubation | TMAO (µg/mL)  After Incubation |
| --- | --- | --- | --- | --- |
| 100% Uremic Serum | 0.072 | N/A | 6.553 | N/A |
| 20% Uremic Serum | BLQ | BLQ | 1.525 | 1.55 |
| 10% Uremic Serum | BLQ | BLQ | 0.762 | 0.82 |
| 5% Uremic Serum | BLQ | BLQ | 0.402 | 0.416 |
| 100% Healthy Serum | 0.04 | N/A | 0.324 | N/A |
| 20% Healthy Serum | BLQ | BLQ | 0.068 | 0.07 |
| 10% Healthy Serum | BLQ | BLQ | 0.036 | 0.035 |
| 5% Healthy Serum | BLQ | BLQ | 0.019 | 0.019 |

Abbreviations: BLQ, below lower limit of quantification; N/A, not applicable.

**S-Figure 1. Specificity of antibodies and range of reactivity.**

**A.**

CYP1A2

AHR

β-ACTIN


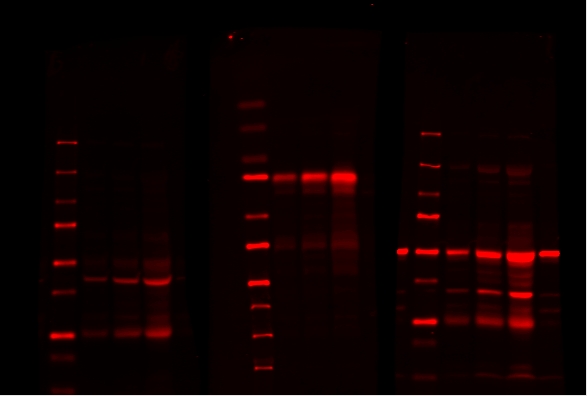


kDa

250

150

100

75

50

37

25

20

15

10

25 μg

50 μg

12.5 μg

25 μg

25 μg

50 μg

12.5 μg

50 μg

12.5 μg

B.

FMO3

β-ACTIN

CYP3A2


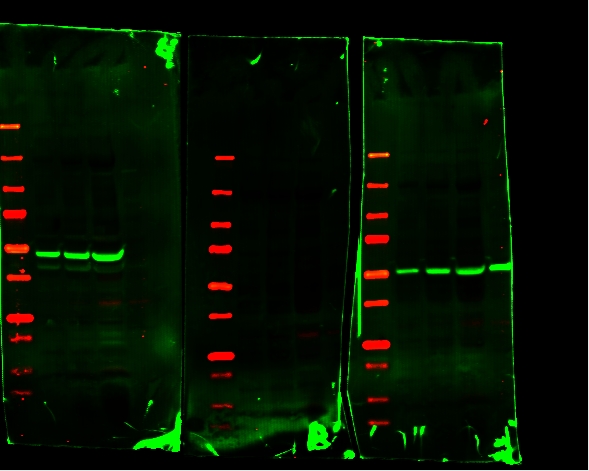


kDa

250

150

100

75

50

37

25

20

15

10

12.5 μg

25 μg

50 μg

50 μg

25 μg

12.5 μg

25 μg

50 μg

12.5 μg

**Figure Legend: S-Figure 1. Specificity of antibodies and range of reactivity.**

The specificity and range of reactivity was assessed for each primary antibody. Specificity was assessed by incubating blots that were loaded with control homogenized rat tissue with each primary antibody to check for overlapping fluorescent bands. Linear ranges of detection were determined for a range (12.5, 25, and 50 µg) of total protein on these blots along with molecular size markers. (A.) Blots were individually incubated with primary antibodies for β-ACTIN, AHR, and CYP1A2, followed by an incubation with a fluorescent donkey anti-mouse secondary antibody. (B.) Blots were individually incubated with primary antibodies for CYP3A2, β-ACTIN, and FMO3, followed by an incubation with a fluorescent donkey anti-rabbit secondary antibody.

**S-Figure 2. Full-length blots for AHR.**

**A.**


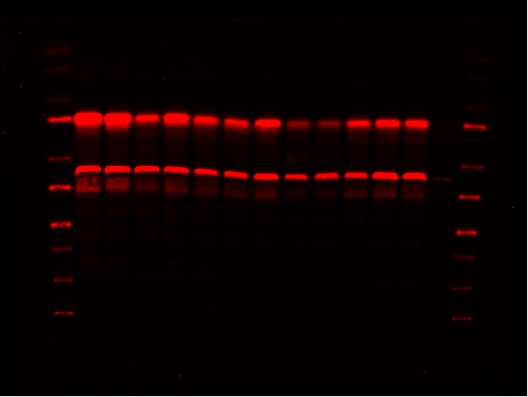


β-ACTIN

kDa

250

150

100

75

50

37

25

20

15

10

AHR

Control

CKD

**B.**


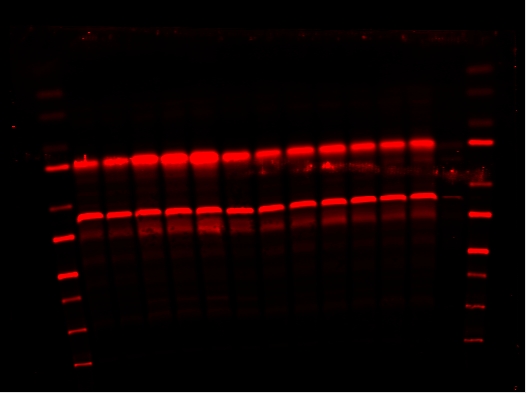


kDa

250

150

100

75

50

37

25

20

15

10

β-ACTIN

AHR

CKD

Control

**Figure Legend: S-Figure 2. Full-length blots for AHR.**

The protein expression of AHR and β-ACTIN were assessed in control (n=12) and CKD (n=12) homogenized rat liver tissues. The n=24 total samples were run on two separate blots shown above in panels (A.) and (B.). (A.) First, individual control (n=6) and CKD (n=6) samples (30 µg total protein) were loaded into the lanes as well as molecular size markers. (A.) represents the blot that is depicted in Figure 2 of the manuscript. (B.) The remaining individual control (n=6) and CKD (n=6) samples (30 µg total protein) were loaded into the lanes as well as molecular size markers. Band intensity of the black and white version of this blot was quantified by densitometry using ImageJ software and normalized to β-Actin expression.

**S-Figure 3. Full-length blots for CYP1A2.**

**A.**


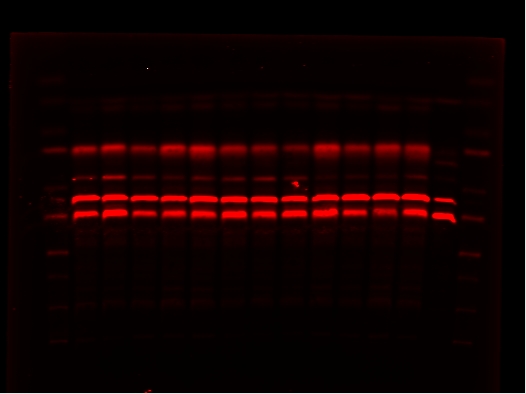


kDa

250

150

100

75

50

37

25

20

15

10

CYP1A2

β-ACTIN

CKD

Control

**B.**


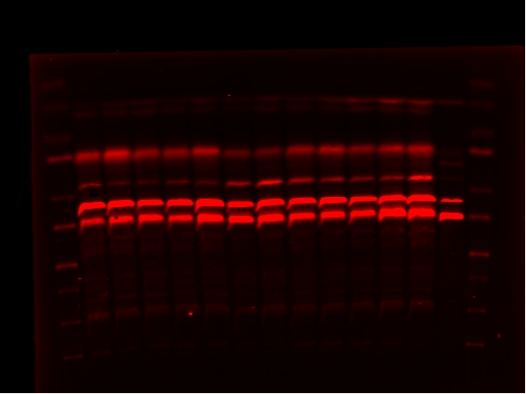


kDa

250

150

100

75

50

37

25

20

15

10

β-ACTIN

CYP1A2

CKD

Control

**Figure Legend: S-Figure 3. Full-length blots for CYP1A2.**

The protein expression of CYP1A2 and β-ACTIN were assessed in control (n=12) and CKD (n=12) homogenized rat liver tissues. (A.) The n=24 total samples were run on two separate blots shown above in panels (A.) and (B.). (A.) First, individual control (n=6) and CKD (n=6) samples (30 µg total protein) were loaded into the lanes as well as molecular size markers. (A.) represents the blot that is depicted in Figure 2 of the manuscript. (B.) The remaining individual control (n=6) and CKD (n=6) samples (30 µg total protein) were loaded into the lanes as well as molecular size markers. Band intensity of the black and white version of this blot was quantified by densitometry using ImageJ software and normalized to β-Actin expression.

**S-Figure 4. Full-length blots for CYP3A2.**

**A.**





kDa

260

160

125

90

75

50

30

25

15

CYP3A2

β-ACTIN

CKD

Control

**B.**





kDa

260

160

125

90

75

50

30

25

15

CYP3A2

β-ACTIN

CKD

Control

**Figure Legend: S-Figure 4. Full-length blots for CYP3A2.**

The protein expression of CYP3A2 and β-ACTIN were assessed in control (n=12) and CKD (n=12) homogenized rat liver tissues. The n=24 total samples were run on two separate blots shown above in panels (A.) and (B.). (A.) First, individual control (n=6) and CKD (n=6) samples (30 µg total protein) were loaded into the lanes as well as molecular size markers. (A.) represents the blot that is depicted in Figure 2 of the manuscript. (B.) The remaining individual control (n=6) and CKD (n=6) samples (30 µg total protein) were loaded into the lanes as well as molecular size markers. Band intensity of the black and white versions of this blot was quantified by densitometry using ImageJ software and normalized to β-Actin expression.

**S-Figure 5. Full-length blots for FMO3.**

**A.**


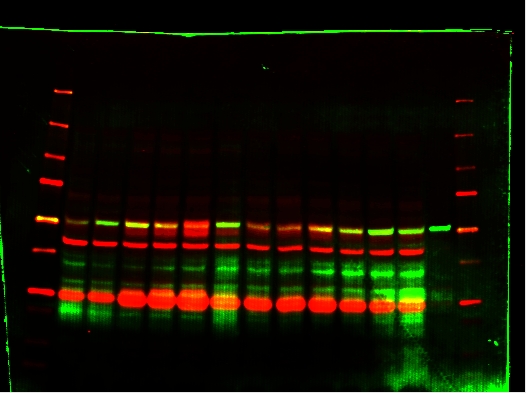


kDa

250

150

100

75

50

37

25

20

15

10

β-ACTIN

FMO3

CKD

Control

**B.**


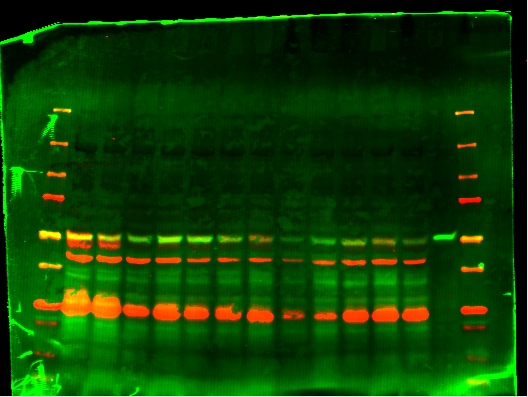


kDa

250

150

100

75

50

37

25

20

15

10

β-ACTIN

FMO3

CKD

Control

**Figure Legend: S-Figure 5. Full-length blots for FMO3.**

The protein expression of FMO3 and β-ACTIN were assessed in control (n=12) and CKD (n=12) homogenized rat liver tissues. The n=24 total samples were run on two separate blots shown above in panels (A.) and (B.). (A.) First, individual control (n=6) and CKD (n=6) samples (30 µg total protein) were loaded into the lanes as well as molecular size markers. (A.) represents the blot that is depicted in Figure 2 of the manuscript. (B.) The remaining individual control (n=6) and CKD (n=6) samples (30 µg total protein) were loaded into the lanes as well as molecular size markers. Band intensity of the black and white versions of this blot was quantified by densitometry using ImageJ software and normalized to β-Actin expression.
